# New Insights into Bioactive Peptides: Design, Synthesis, Structure–Activity Relationship

**DOI:** 10.3390/ijms252312922

**Published:** 2024-12-01

**Authors:** Flavia Anna Mercurio, Marilisa Leone

**Affiliations:** Institute of Biostructures and Bioimaging (IBB), National Research Council of Italy (CNR), Via Pietro Castellino 111, 80131 Naples, Italy; flaviaanna.mercurio@cnr.it

In recent decades, peptides have attracted significant attention not only from Academia but also from big Pharma as novel potential therapeutic compounds [1]. Peptides are made up of amino acids and only differ from proteins in terms of their smaller size (up to ~50 residues). Contrarily to small molecules, peptides do not generally obey to “Lipinski’s rule of five” (also known as “Pfizer’s rule of five”) for orally bioactive drugs [2]. However, medicinal chemistry efforts have lately allowed researchers to establish novel synthetic routes so that peptide drug-like features can now be largely improved. For example, peptides translated into peptidomimetics or peptoids with unusual backbones, unnatural amino acids or peculiar cyclic organizations can be provided with specific secondary structure elements and become more resistant to proteolytic cleavage or even acquire better membrane permeability, resulting in improved drug-like characteristics for therapeutic applications [1,3]. Despite their poor pharmacokinetics, in comparison with small molecules, peptides are characterized by great pharmacodynamic properties. In fact, peptides can be designed ad hoc to modulate specific “undruggable” protein–protein interactions and achieve desired in-cell biological effects. Their larger size in comparison with small molecules allows peptides to establish many different interactions with target macromolecules and achieve high affinity, specificity and efficacy that produce fewer side- and off-target effects for applications in the therapeutic field [2]. Peptides also have some advantages with respect to other therapeutic biologics (like antibodies, therapeutic proteins and vaccines), including decreased immunogenicity and lower production-associated costs [4]. In the biomedical field, peptides can be employed as original therapeutics but also be implemented as drug-delivery tools to distribute drugs to specific cells that overexpress certain receptors on their surfaces, or as theragnostic compounds, for example, by exploiting their self-assembly properties and/or linking them to contrast agents for employment in magnetic resonance imaging.

Considering primarily the impact of peptides in the biomedical field and the variety of their applications, in the framework of this Special Issue, we invited authors to submit research and review articles centered on the design and characterization of bioactive peptides, with a particular focus on crucial structure–activity data that could promote the understanding of their mechanisms of action within the cell.

This Special Issue includes 10 research and 2 review articles.

The development of therapeutic peptides for treating several pathologies like viral and bacterial infections, cancer and obesity is presently ongoing [5]. Peptides are of great interest in the field of anticancer drug discovery as, due to their target specificity, they can eventually be employed to set up original therapeutic routes with fewer side effects for cancer patients, which is particularly worthwhile considering the health-related risks connected with chemotherapy drugs [6].

Most of the works included in this Special Issue are centered on peptides with anticancer properties. Yurkina et al. [7] explores peptides deriving from the proinflammatory cytokine TNF (Tumor Necrosis Factor) and its receptor TNFR1 (Tumor Necrosis Factor Receptor 1) as possible therapeutics in antitumor or anti-cytokine treatments. The interaction between TNF and TNFR1 activates a two-stage cytotoxic pathway which determines tumor cell death. A structure-based design of three peptides encompassing the TNF and TNFR1 reciprocal binding interfaces was achieved with the aim to modulate the TNF cytotoxic signal transduction. Analyses by microscale thermophoresis, confocal microscopy and cytotoxicity assays on L929 mouse fibroblast cells led to the identification of a peptide derived from TNFR1 that can compete with the binding with its natural ligands and a peptide designed from TNF which is able to bind to TNFR1 and induce the activation of cytotoxic signal transduction [7].

The contribution by Biniari et al. [8] reports instead on promising compounds as possible anticancer candidates for further in vitro and in vivo pharmacological investigations. Gonadotropin-releasing hormone (GnRH) is a decapeptide which regulates human reproduction and fertility through the production of gonadotropins. Gonadotropin-releasing hormone receptor type I (GnRHR I) is a GnRH receptor which is overexpressed on the cell surface of hormone-dependent cancers, such as ovarian and endometrial tumors, and thus is a key target for the development of cancer therapeutics [8]. The GnRH peptide in association with cytotoxic agents can be used as a vector for targeted drug delivery. Thus, in order to allow the selective release of cytotoxic agents in cancer cells, the authors designed and analyzed a series of anthraquinone/mitoxantrone–GnRH conjugates with high binding affinities for GnRH I [8].

Bleomycins are a glycopeptide congener family and are commercialized as Bleoxane. Bleoxane is used as an antitumor antibiotic for the treatment of diseases such as squamous cell carcinoma and malignant lymphoma. The interactions of several bleomycins with human serum albumin (HSA) and human acid glycoprotein (AGP), two plasma proteins involved in drug delivery, was investigated in the study by Longo and colleagues [9]. Several spectroscopic techniques, such as circular dichroism, UV-vis absorbance and fluorescence, were implemented to evaluate protein–bleomycin interactions. The results indicated that bleomycins bind AGP with higher affinity than HAS [9]. This information is important for in vivo pharmacokinetic studies. In addition, considering that AGP is overexpressed in cancer patients, the bleomycin dosage used in cancer therapies could be regulated according to its high affinity to this protein [9].

Two other bioactive peptides, bradykinin (BK) and neurotensin (NT), were chemically modified and analyzed for their activities in colorectal cancer cells (CRCs) in the study of Szaryńska et al. [10]. The effect on cell viability of BK, NT and their analogs was investigated on different CRC lines. The influence of the novel peptide analogs on cancer stem cell (CSC) spherogenic potential and phenotype was also evaluated. The results indicated that a group of these peptides influenced all the observed cellular features, while a second group was most effective in reducing the number of CSCs with a parallel extensive decrease in CRC viability. These BK and NT analogs could thus be further tested to better understand their general anticancer potential [10].

This Special Issue also includes a contribution from our Laboratory [1]. We present a review article summarizing in silico approaches specifically developed for the drug discovery of therapeutic bioactive peptides. Computational tools can be very useful in optimizing the costs and time spent for drug discovery campaigns. These tools can be used to generate large libraries of virtual peptides that can be preselected by means of different strategies, mainly structure-based virtual screening (SBVS) docking approaches, for further experimental validation. Our review also describes examples reported in the literature in which not only anticancer but also antimicrobial/antiviral peptides and peptides to be used against amyloid fiber formation have been designed in silico [1].

A few studies reported within this Special Issue investigate bioactive peptides as therapeutics for diseases other than cancer. For example, Zhu et al. [11] analyzed the effect of oligopeptides (GOPs) isolated from Panax ginseng C. A. Meyer on cell fate stability, which is connected to age-related pathologies. Cell viability and biochemical assays indicated that GOP could suppress oxidative stress and protect mitochondrial function by increasing cell viability, preventing the cell cycle arrest and improving telomerase activity. GOPs also had positive effects on cell stability in PC-12 neuronal cells by inhibiting autophagic cell death [11]. The beneficial effects of GOPs on cellular processes could be applied in diverse research fields.

Wang and collaborators report on anti-thrombotic and analgesic peptides in their research article [12]. The peptide poeciguamerin, a serine protease inhibitor including an antistasin-like domain, was isolated from the salivary gland secretion of *P. manillensis* leech and its amino acid sequence and biological activity were investigated. This peptide was found to exert analgesic effects by modulating elastase activity and to have anticoagulant properties by inhibiting the pathways involving FXIIa (Factor XIIa) and kallikrein. Porciguamerin can therefore be further optimized to develop novel bioactive peptides for the treatment of pain and thrombosis after surgery or in inflammatory conditions [12].

Another attractive field of peptide research concerns antimicrobial peptides (AMPs) that due to the SARS-CoV-2 (Severe Acute Respiratory Syndrome Coronavirus 2) pandemic and the wide spread of different viral and bacterial infections have recently captured the attention of the scientific community. Contributions to this topic are also included in this Special Issue. In the study of Bugatti et al. [13], a structure-based approach was conducted to design a few peptidomimetics (PMs) that were then tested as potential inhibitors of the interaction between the SARS-CoV-2 spike receptor-binding domain (S-RBD) and the human ACE2 (Angiotensin-converting enzyme 2) receptor. A few of the novel-generated PMs showed low-micromolar-dissociation constants in surface plasmon resonance experiments against SARS-CoV-2 S-RBD, and one of them was found to exert a slight inhibition of SARS-CoV-2 entry in human cell lines expressing the ACE2 receptor [13].

Similarly, the review by Vincenzi et al. [1] also dedicated a section to publications in which drug discovery in silico approaches were aimed to find antimicrobial peptides against SARS-CoV-2 infection.

The second review article included in this Special Issue summarizes the limitations and state-of-the-art progress in the application of antimicrobial peptides for the treatment of atopic dermatitis (AD) [14]. This chronic inflammatory disease of the skin has various environmental, bacterial and genetic causes, which compromise the function of the skin barrier. The most common comorbidities related to AD are skin infections, which are treated with chronic therapies with undesired secondary effects. AMPs are thus a promising therapeutic alternative for the treatment of AD due to their potential to act on the skin microbiome, fight infections and modulate the skin immune response [14].

One of the research articles [15] has a somehow indirect connection to the field of therapeutic peptides as it presents a purification route for fluorinated therapeutic compounds. Fluorinated drug molecules and peptides with fluorinated amino acids are gaining pharmaceutical importance due to favorable features such as improved pharmacokinetic and physicochemical properties. Nonetheless, the commercial availability of fluorinated drugs is restricted due to the scarcity of chromatographic methods to obtain pure enantiomers. In this context, a chromatographic protocol was explored to achieve enantioselective separations of fluorine-substituted tryptophan analogs by the application of macrocyclic glycopeptide-based selectors covalently joined to core–shell particles [15]. This work could be considered as a basis for further improvements in the enantioseparation of fluorinated amino acids and could contribute to the advancement of the field of fluorinated peptides for therapeutic applications.

The remaining research articles published in this Special Issue do not concern bioactive peptides relevant to human health. In fact, the study by Chinta et al. [16] explores the effect of bioactive peptides in pest control. Insect neuropeptides and their receptors are considered important targets for pest management as they control almost all physiological processes related to insect life phases. In a previous work, the authors had developed a novel technology, Receptor-interference (Receptor-i), in which libraries of phage-displayed peptides were screened against the pheromone biosynthesis activating neuropeptide receptor (PBAN-R) of the red imported fire ant (*Solenopsis invicta*) [17]. In the study published in this Special Issue, a few of the peptides previously selected with the Receptor-i technology were further analyzed with small-scale feeding bioassays to determining the mortality of fire ant workers. The results confirmed the relevance of Receptor-i, which can be further applied to other agricultural and medical pests [16].

The self-assembly of peptides into amyloid fibers represents an attractive field of study for the scientific community, mainly in relation to neurodegenerative disorders like Alzheimer’s disease [1]. An interesting work presented within this Special Issue reports on the application of amyloid fibril (AF) modulation in the food industry [18]. In this study, the effect of different NaCl concentrations on the formation of AFs in cooked wheat noodles was investigated. Several techniques were used to identify AFs and analyze them in terms of different properties (like molecular weight distribution, hydrophobicity of surface, morphology and secondary structure). The results indicated that NaCl can affect the formation and growth of AFs, and therefore the functional properties of gluten proteins in wheat flour products [18].

In conclusion, we believe that this Special Issue represents an inspirational collection of studies that can be consulted by researchers involved in very diverse aspects of peptide science. Most contributions describe the development of peptides as potential therapeutic agents, especially in the anticancer field [1,7,8,10], but articles and review sections focused on bioactive peptides working as antimicrobials are also included [1,13,14]. Although most of the contributions to this Special Issue concern peptide applicative examples, a more technical study related to the set-up of a purification strategy that could be useful to scientists interested in the synthesis of fluorinated peptides is also presented [15]. On a different note, researchers involved in the fields of food chemistry and peptides/proteins forming amyloid fibers or in the employment of peptides for pest control will also find useful information within this collection of studies [16,18].

We anticipate that in the near future, thanks to the introduction of clever Artificial Intelligence instruments [19,20,21,22] currently revolutionizing the structural biology field, we will witness tremendous advancements in in silico peptide design tools that, along with further developments in synthetic medicinal chemistry routes, will lead to a large increase in therapeutic peptides on the market.
